# Clinical and genomic determinants of extraordinary response and resistance to androgen receptor pathway inhibitors in men with metastatic androgen pathway modulator sensitive (APMS) prostate cancer

**DOI:** 10.1038/s41391-026-01145-0

**Published:** 2026-08-05

**Authors:** Jane McKenzie, Lauren Howard, Joseph J. Park, Bilal Ashraf, Tara Seibert, Kallie White, Sundhar Ramalingam, Jeffrey W. Shevach, Michael R. Harrison, Christopher Hoimes, Matthew Labriola, Jordan Infield, Dillon Cockrell, Hannah D. McManus, Daniel J. George, Andrew J. Armstrong

**Affiliations:** 1 Duke Cancer Institute Center for Prostate and Urologic Cancers, Department of Medicine, Division of Medical Oncology, Duke University, Durham, NC 27710 USA; 2 Department of Biostatistics, Duke University, Durham, NC USA

**Keywords:** Cancer genetics, Prostate cancer, Cancer therapy, Prognostic markers, Prostate cancer

## Abstract

**Background:**

Identifying clinical and genomic predictors of response to androgen deprivation therapy (ADT) and androgen receptor pathway inhibitors (ARPI) for metastatic androgen pathway modulator sensitive (APMS) prostate cancer is essential for improving therapeutic decision-making. Divergent outcomes are observed in practice, with some achieving exceptional responses (ER) while others experience early resistance or non-response (NR), the biologic basis of which remains unclear.

**Methods:**

A retrospective review was conducted of patients with APMS prescribed ADT/ARPI from 2015–2024. ER and NR were defined by upper ( > 53.36 months) and lower ( < 10.41 months) quartiles of progression-free survival (PFS). Objectives were to associate baseline genomic and clinical factors with ER/NR status, PFS, overall survival (OS) and PSA kinetics..

**Results:**

Among 3363 prescribed ADT/ARPI, 224 were treated for APMS, with 154 (69%) undergoing temporal molecular testing. Alteration of ≥1 tumor suppressor gene with or without proliferative gene co-alteration was associated with NR while no single alteration predicted achieving ER. ECOG < 1, lack of pain, low-volume disease, higher hemoglobin and albumin, and normal alkaline phosphatase were associated with improved PFS, OS, and PSA nadir <0.2 ng/mL at 12 months. Alterations in *TP53* (HR 1.60 [95%CI 1.02–2.50], *p* = 0.039; HR 1.91 [95%CI 1.05-3.49], *p* = 0.035) and *MYC* (HR 2.01 [95%CI 1.03-3.91], *p* = 0.040; HR 3.81 [95%CI 1.81-7.99], *p* < 0.001) were associated with inferior PFS and OS, while *BRCA2* (HR 2.12 [95%CI 1.02–4.42], *p* = 0.045) additionally associated with PFS, and *RAD21* (HR 2.55 [95%CI 1.13–5.77], *p* = 0.024) with OS. On multivariable analysis, *MYC* gain associated with OS (HR 4.41 [95%CI 1.24-15.7], *p* = 0.022). Pathway-level analysis showed proliferative gene alterations were associated with failure to achieve PSA nadir <0.2 ng/mL at 12 months (HR 0.49 [95%CI 0.24, 0.98], *p* = 0.047).

**Conclusions:**

In this real-world APMS cohort, we identified pre-treatment clinical and genomic predictors of patient benefits and long-term survival with ADT/ARPI therapy.

## Background

Prostate cancer remains the second most diagnosed cancer worldwide and the second leading cause of cancer-related death in men in the United States [1]. Following proven survival benefits for patients with metastatic androgen pathway modulator resistant (APMR) prostate cancer, androgen deprivation therapy (ADT) in combination with androgen receptor pathway inhibitors (ARPI), with or without docetaxel, is standard-of-care for patients with metastatic androgen pathway modulator naïve/sensitive prostate cancer (mAPMN or mAPMS), using PCWG4-preferred terminology [2, 3]. Median overall survival (OS) rates now exceed 4 years in seminal clinical trials [4–6], while for high-volume APMS, adding ARPI to ADT/docetaxel further improves survival [6–8].

A minority of patients exhibit minimal response to first-line ARPI, with primary resistance rates of 4–7% for mAPMS [9, 10]. Conversely, some experience prolonged clinical benefit. This proportion is less well-characterized, however long-term follow-up from randomized trials report 22–33% of patients continue ARPI after up to 8 years [11–13]. Real-world data suggest enduring responses are less common in practice, with <25% of patients in a US Veterans Affairs cohort remaining on ARPI at 2.8 years [14].

Current prognostic models center around clinical factors including disease volume, de novo metastatic disease, visceral metastases and Gleason score [15–18], though genetic biomarkers are increasingly recognized as prognostic and predictive. Aggressive mAPMS phenotypes often harbor loss of function mutations in tumor suppressor genes (TSG), including *TP53, PTEN and RB1*, and alterations in *MYC* and cell cycle pathway genes, which are strongly associated with early resistance [19–21]. In contrast, alterations in *SPOP* and the WNT pathway have been associated with favorable prognosis [20]. Integrating genetic biomarkers may enable more personalized mAPMS management, guiding treatment intensification for patients with poor prognostic disease or deintensification strategies for lower-risk patients to balance the long-term risks and burden of treatment.

This study sought to describe the clinical and molecular landscape of patients with APMS receiving ARPI and analyze the clinical and genomic predictors of exceptional response and non-response.

## Methods

### Study population

A physician-abstracted retrospective review of Duke Health patients prescribed ARPI between January 2015 to January 2024 was matched to an institutional molecular testing database capturing germline and somatic testing. Clinicopathologic variables were extracted from the electronic medical record. Concurrent systemic therapies other than docetaxel were excluded. Progression-free survival (PFS) and OS were defined from ARPI initiation to progression or death, respectively. Progression was defined as; (i) rising PSA per Prostate Cancer Working Group 3 criteria [22], (ii) radiographic progression, or (iii) clinician-determined clinical progression. Disease volume was defined per CHAARTED criteria [23]. Aggressive-variant prostate cancer (AVPC) was defined as; (i) presence of liver metastases, (ii) bulky radiographic disease ( > 2 cm short axis lymph nodes or >1 cm long axis visceral metastases) with low serum PSA ( < 10 ng/ml), (iii) elevated serum LDH, CEA or chromogranin A levels, or (iv) histologically-confirmed neuroendocrine differentiation. Exceptional responders (ER) and non-responders (NR) were defined using Kaplan-Meier-derived PFS quartiles - ER by the upper quartile ( ≥ 75^th^ percentile) and NR by the lower quartile ( ≤ 25^th^ percentile).

### Molecular alterations

Testing included tissue-based next-generation sequencing (FoundationOne CDx assay) or cell-free DNA assays (cfDNA; Guardant360 assay). Temporal definitions for molecular testing were defined as prior to or within 3 months of ARPI initiation for non-responders and prior to or within 6 months of ARPI initiation for exceptional responders, selected to maximize patient inclusion whilst minimizing confounding from treatment-emergent alterations acquired during disease progression [24]. Only pathogenic or likely pathogenic alterations (tier 1-2) were analyzed; variants of undetermined significance were excluded. Alterations were further grouped by biologic pathway (Supplementary Methods).

### Statistical considerations

The primary objective was to evaluate associations between baseline molecular and clinical factors with ER/NR status. Secondary objectives included describing baseline molecular and clinical landscape of APMS and assessing associations with PFS, OS and PSA kinetics.

Patient characteristics were described in the overall APMS cohort (*n* = 224). Among those with temporal molecular testing (*n* = 154), univariable analysis using Cox proportional hazards models assessed associations between baseline characteristics, disease characteristics, and top 10 most common genetic alterations and the risk of PFS and OS. Covariates were included in the multivariable model based on established clinical rationale and association on univariable analysis. Variables with a high level of missing data (such as some laboratory values) were not included in multivariable models. Associations between molecular alterations/pathways and ER/NR status were tested using Wilcoxon rank sum test for continuous measures and Fisher’s exact test for categorical variables. Logistic regression assessed predictors of PSA nadir <0.2 ng/mL within 12 months and post-progression exploratory analyses. Per ER/NR status, a Kaplan-Meier curve evaluated PFS, OS and post-progression OS.

### Ethics approval and consent to participate

The Duke Health Institutional Review Board approved the protocol, which was conducted in compliance with the principles of the Declaration of Helsinki and guidelines for Good Clinical Practice. This study was determined to be informed consent exempt. All methods were performed in accordance with these guidelines.

## Results

Among 3363 patients prescribed an ARPI during the 9-year study period, 749 (22%) had tumor molecular data registered and 224 met inclusion criteria (Supplementary Fig. S1). Baseline clinicopathologic factors are reported in Table 1. Median age was 68 years (range 42–89). De novo metastatic disease occurred in 177 (80%) and 145 (65%) had high volume disease, including 14 (6.3%) with liver metastases. Median baseline PSA prior to ADT was 73 ng/mL (range 0-46,242). Eighty (36%) patients received triplet therapy with docetaxel (median 6 cycles [range 1–9]). Thirty-seven (16.5%) received radiation to prostate primary while 22 (9.8%) received metastasis-directed therapy with radiotherapy.

**Table 1:** Baseline patient and disease characteristics of APMS patients (*N* = 224).

Patient characteristics	
Baseline age	68 (42–89)
Race	
Another race	10 (4.5%)
Black or African American	53 (24%)
White	161 (72%)
History of tobacco smoking	118 (53%)
ECOG	
1-3	117 (52%)
0	83 (37%)
Unknown	24 (11%)
**Disease characteristics**	
Gleason score	
6-7	36 (16%)
8-10	139 (62%)
Unknown	49 (22%)
PSA (ng/mL)	73 (0 – 46242)
De novo metastatic disease	177 (80%)
Pain at ARPI initiation	96 (47%)
Volume of disease	
High	145 (65%)
Low	79 (35%)
Metastasis locations	
Any visceral	44 (20%)
Bone and lymph node	112 (50%)
Bone only	36 (16%)
Lymph node only	32 (14%)
Liver metastases	14 (6.3%)
Median hemoglobin (g/dL)	13.30 (5.90 – 18.50)
Median albumin (g/dL)	3.90 (1.80 – 5.00)
Alkaline phosphatase > ULN (U/L)	106 (60%)
Neutrophil to lymphocyte ratio	2.72 (0.53 – 19.50)
**Treatment characteristics**	
First-line ARPI agent used	
Enzalutamide	49 (22%)
Abiraterone	123 (55%)
Darolutamide	44 (20%)
Apalutamide	8 (3.6%)
Prior local therapy*	49 (22%)
Triplet therapy	80 (36%)
Months from genetic test to ARPI initiation	1 (-77 – 123)

^1^ Median (Min - Max) n (%), *radical prostatectomy or prostate radiation.

### Baseline molecular characteristics

**Fig. 1:**
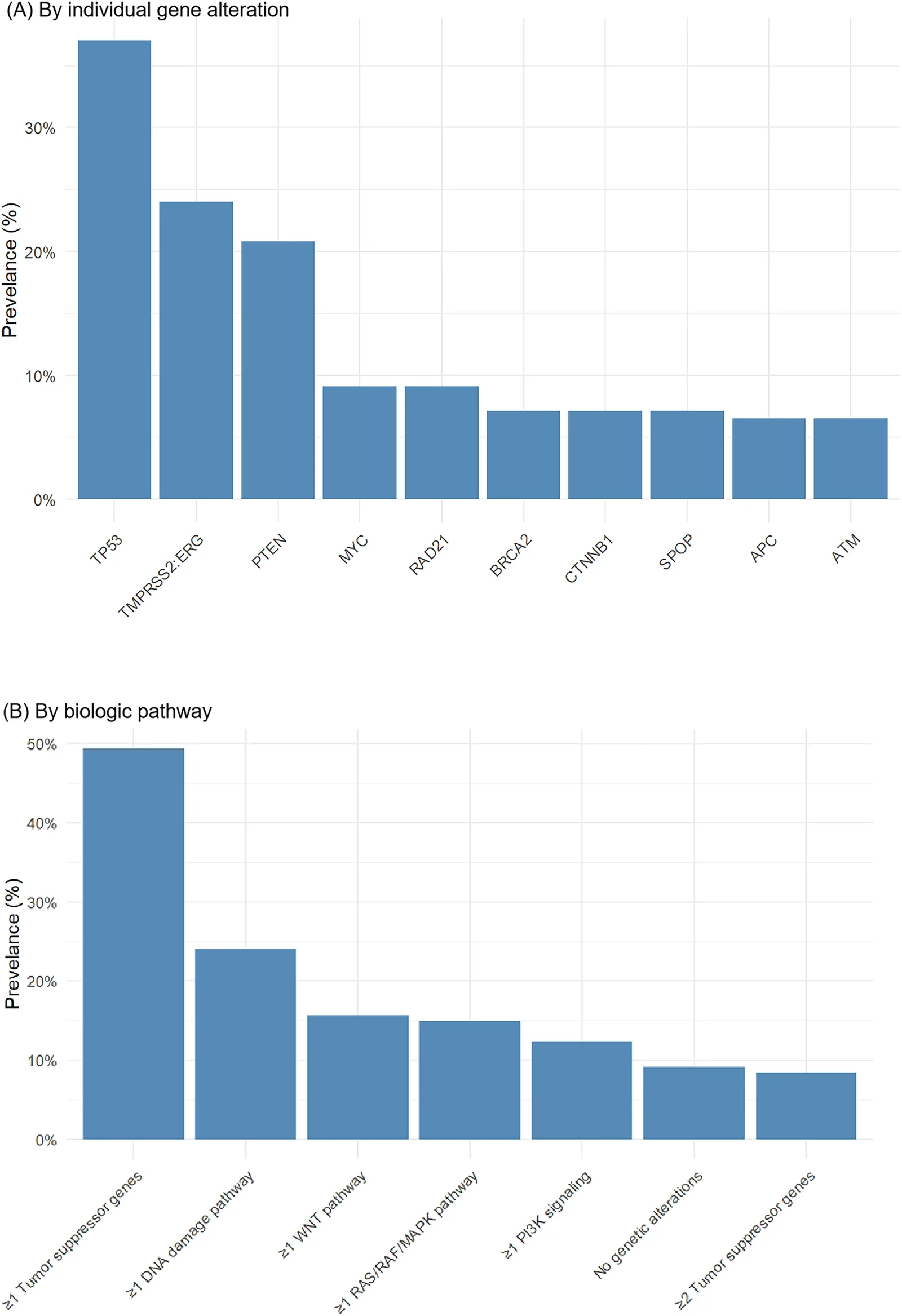
Prevalence of genomic alterations across mAPMS samples (*N* = 154). **A** By individual gene. **B** By biologic pathway.

Of 224 mAPMS patients, 154 (68.7%) had molecular sequencing temporal to ARPI initiation (median 1 month prior). The majority (81%) had tissue-based FoundationOne CDx NGS testing (102 primary tissue, 22 metastatic tissue), while 30 (19%) had cfDNA testing with Guardant360 assay. The most common baseline alterations were in *TP53* (37%), *TMPRSS2::ERG* (24%) and *PTEN* (21%) (Fig. 1A), with a median of 3 (interquartile range [IQR] 1-4) total alterations. By biologic pathway, proliferative genes (69%) were most altered, followed by *TSG* (49%) and DNA damage pathway genes (24%) (Fig. 1B). An Oncoprint demonstrates alterations occurring in >1 patients and co-alteration patterns (Supplementary Fig. S2).

### Exceptional responder/Non-responder analysis

Exceptional responders (ER) were defined by the upper PFS quartile (achieving PFS > 53.36 months) while short-term or non-responders (NR) were defined by the lower quartile ( < 10.41 months). Overall, 23 (10.6%) achieved ER, 56 (25%) were NR, and 145 (64.7%) had intermediate responses (i.e. not ER or NR). Median time to PSA progression was 7 months (95% CI 4.8–9.3) for NR, 46 months (95% CI 36–53) for intermediate responders, and not reached for ER. Despite short-term response, PSA50 and PSA90 rates for NR were 96% and 84%, respectively, versus both 100% for ER. However, only 21% of NR achieved PSA nadir <0.2 ng/mL within 12 months compared to 100% of ER, which significantly associated with ER status (OR 5.25, 95% CI 1.32–22.7). Expectedly, PFS and OS were longer for ER versus NR patients (Supplementary Fig. S3).

**Fig. 2:**
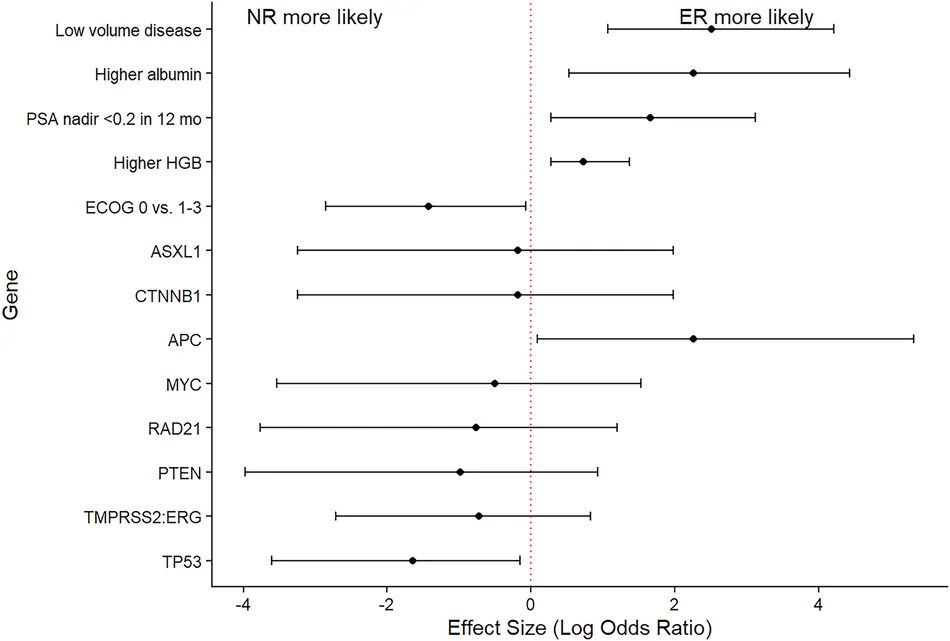
Forest plot of top clinical and genetic factors associated with ER status (log odds 95% CI) (*N* = 46).

ER patients were more likely to have low-volume disease (OR 12.4, 95% CI 2.93–67.9) and higher hemoglobin (OR 2.08, 95% CI 1.32–3.95) and albumin (OR 9.60, 95% CI 1.70–84.4), while ECOG ≥ 1 was associated with NR status (OR 0.24, 95% CI 0.06–0.93) (Fig. 2)

ER patients had a lower genomic alteration burden (Table 2), with no ER patients having >2 genes altered. NR patients were significantly enriched for *TP53* (48% NR vs. 15% ER, *p* = 0.038) and TSG alterations (58% NR vs. 15% ER, *p* = 0.010). *APC* alterations were numerically more common in ER (3% vs. 23%, *p* = 0.062) (Fig. 2). Analysis of *SPOP* alterations was limited due to small numbers (2 (6.1%) in NR, 1 (7.7%) in ER). Co-occurrence of ≥1 TSG and ≥1 proliferative gene alterations was most strongly associated with NR (58% vs. 7.7%, *p* = 0.002) (Table 2).

**Table 2:** Univariable association between gene mutations, PSA response and exceptional response status.

	PSA nadir < 0.2 within 12 months					Exceptional response status		
Characteristic	Counts		PSA nadir < 0.2 vs ≥ 0.2			Counts		NR vs. ER
	PSA nadir ≥ 0.2 N = 73^*1*^	PSA nadir < 0.2 N = 81^*1*^	OR^*2*^	95% CI^*2*^	*p*-value	NR N = 33^1^	ER N = 13^1^	*p*-value^3^
**Burden of genetic alterations**								
Total genes altered	3 (2, 4)	3 (1, 4)	0.95	0.84, 1.07	0.4	4 (2, 5)	2 (1, 2)	**<0.001**
Total genes altered								**<0.001**
<3 genes	50 (68%)	53 (65%)	—	—		6 (48%)	13 (100%)	
≥3 genes	23 (32%)	28 (35%)	1.15	0.59, 2.26	0.7	17 (52%)	0 (0%)	
**Genomic mutations**								
*TP53*	27 (37%)	30 (37%)	1.00	0.52, 1.93	>0.9	16 (48%)	2 (15%)	**0.038**
*TMPRSS2I:ERG*	24 (33%)	13 (16%)	0.39	0.18, 0.83	**0.016**	9 (27%)	2 (15%)	0.5
*PTEN*	18 (25%)	14 (17%)	0.64	0.29, 1.39	0.3	6 (18%)	1 (7.7%)	0.7
*MYC*	6 (8.2%)	8 (9.9%)	1.22	0.40, 3.89	0.7	4 (12%)	1 (7.7%)	>0.9
*RAD21*	6 (8.2%)	8 (9.9%)	1.22	0.40, 3.89	0.7	5 (15%)	1 (7.7%)	0.7
*SPOP*	4 (5.5%)	7 (8.6%)	1.63	0.47, 6.46	0.5			
*BRCA2*	5 (6.8%)	6 (7.4%)	1.09	0.31, 3.93	0.9	5 (15%)	0 (0%)	0.3
*CTNNB1*	5 (6.8%)	6 (7.4%)	1.09	0.31, 3.93	0.9	3 (9.1%)	1 (7.7%)	>0.9
*APC*	3 (4.1%)	7 (8.6%)	2.21	0.59, 10.6	0.3	1 (3.0%)	3 (23%)	0.062
*ATM*	3 (4.1%)	7 (8.6%)	2.21	0.59, 10.6	0.3			
*PIK3CA*						4 (12%)	0 (0%)	0.3
*ASXL1*						3 (9.1%)	1 (7.7%)	>0.9
**Genomic pathways**								
≥1 Tumor suppressor gene	38 (52%)	38 (47%)	0.81	0.43, 1.53	0.5	19 (58%)	2 (15%)	**0.010**
≥2 Tumor suppressor genes	7 (9.6%)	6 (7.4%)	0.75	0.23, 2.38	0.6	3 (9.1%)	1 (7.7%)	>0.9
≥1 WNT pathway	10 (14%)	14 (17%)	1.32	0.55, 3.26	0.5	5 (15%)	4 (31%)	0.2
≥1 DNA damage pathway	17 (23%)	20 (25%)	1.08	0.51, 2.29	0.8	12 (36%)	2 (15%)	0.3
≥1 RAS/RAF/MAPK pathway	8 (11%)	15 (19%)	1.85	0.75, 4.86	0.2	8 (24%)	0 (0%)	0.084
≥1 PI3K signaling	13 (18%)	6 (7.4%)	0.37	0.12, 0.99	0.057	8 (24%)	0 (0%)	0.084
DNA damage sensing/repair	17 (23%)	20 (25%)	1.08	0.51, 2.29	0.8	12 (36%)	2 (15%)	0.3
Proliferative genes	56 (77%)	50 (62%)	0.49	0.24, 0.98	**0.047**	29 (88%)	9 (69%)	0.2
Epigenetic genes	11 (15%)	8 (9.9%)	0.62	0.23, 1.62	0.3	6 (18%)	1 (7.7%)	0.7
TSG & DNA repair	7 (9.6%)	8 (9.9%)	1.03	0.35, 3.10	>0.9	6 (18%)	1 (7.7%)	0.7
TSG & Proliferative	31 (42%)	28 (35%)	0.72	0.37, 1.37	0.3	19 (58%)	1 (7.7%)	**0.002**
TSG & Epigenetic	4 (5.5%)	2 (2.5%)	0.44	0.06, 2.31	0.3	3 (9.1%)	0 (0%)	0.5
DNA repair & Proliferative	12 (16%)	16 (20%)	1.25	0.55, 2.91	0.6	10 (30%)	1 (7.7%)	0.14
DNA repair & Epigenetic	5 (6.8%)	3 (3.7%)	0.52	0.10, 2.21	0.4	4 (12%)	0 (0%)	0.3
Proliferative & Epigenetic	9 (12%)	4 (4.9%)	0.37	0.10, 1.19	0.11	6 (18%)	0 (0%)	0.2
No genetic alterations	3 (4.1%)	11 (14%)	3.67	1.09, 16.7	0.054	1 (3.0%)	1 (7.7%)	0.5

^1^Median (Q1, Q3); *n* (%), ^2^ *OR* Odds Ratio, *CI* Confidence Interval, ^3^ Wilcoxon rank sum test/Fisher’s exact test.

*TSG* = tumor suppressor gene.

### Survival outcomes

In the overall mAPMS cohort, median follow-up was 48 months (95% CI 40–56). Median PFS was 28 months (IQR 10–53), with a median time to AMPR of 30 months (IQR 12–62). Median OS was 56 months (IQR 30–92).

In the temporal molecular testing subset (*n* = 154), inferior PFS was observed in Black patients (HR 1.86, 95% CI 1.18–2.94), tobacco use (HR 1.64, 95% CI 1.05–2.56), and liver metastases (HR 2.47, 95% CI 1.30–4.67), while ECOG ≥ 1 associated with both poorer PFS (HR 3.18, 95% CI 1.89–5.34) and OS (HR 3.10, 95% CI 1.48–6.51). Disease factors associated with improved PFS and OS included a lack of pain (PFS HR 0.37, 95% CI 0.23–0.58; OS HR 0.32, 95% CI 0.17–0.62), low-volume disease (PFS HR 0.35, 95% CI 0.21–0.59; OS HR 0.34, 95% CI 0.16–0.71), higher hemoglobin (PFS HR 0.72, 95% CI 0.66–0.80; OS HR 0.74, 95% CI 0.66–0.84) and albumin (PFS HR 0.39, 95% CI 0.25–0.60; OS HR 0.50, 95% CI 0.27–0.93), and normal alkaline phosphatase (PFS HR 0.46, 95% CI 0.29-0.72; OS HR 0.41, 95% CI 0.21-0.78) (Table 3). De novo metastatic presentation was not associated with either PFS or OS.

**Table 3:** Univariable and multivariable clinical and genomic features associated with survival outcomes (*N* = 154).

	Characteristic	PFS HR (95% CI)	*p*-value	OS HR (95% CI)	*p*-value
**Univariable analysis**	Age	0.99 (0.97-1.02)	0.4	1.00 (0.97 – 1.04)	0.9
	Race				
	White	-	-	-	-
	Black/African Amer.	1.86 (1.18-2.94)	**0.008**	1.57 (0.37-6.69)	0.5
	Other	0.71 (0.17-2.92)	0.6	1.61 (0.85-3.04)	0.14
	History of smoking	1.64 (1.05-2.56)	**0.030**	1.32 (0.72-2.41)	0.4
	Gleason Score				
	6-7	-	-	-	-
	8-10	0.88 (0.48-1.63)	0.7	0.83 (0.36-1.93)	0.7
	Unknown	1.62 (0.82-3.20)	0.2	1.27 (0.49-3.29)	0.6
	ECOG				
	0	-	-	-	-
	1-3	3.18 (1.89-5.34)	**<0.001**	3.10 (1.48-6.51)	**0.003**
	Unknown	3.68 (1.37-9.90)	**0.010**	2.74 (0.59-12.7)	0.2
	Lack of pain at ARPI initiation	0.37 (0.23-0.58)	**<0.001**	0.32 (0.17-0.62)	**<0.001**
	De novo metastatic	1.36 (0.75-2.47)	0.3	0.80 (0.39-1.62)	0.5
	Low volume disease	0.35 (0.21-0.59)	**<0.001**	0.34 (0.16-0.71)	**0.004**
	Liver metastases	2.47 (1.30-4.67)	**0.006**	1.94 (0.85-4.42)	0.11
	Metastasis locations				
	Bone only	-	-	-	-
	Lymph Node only	0.31 (0.11-0.86)	**0.025**	1.25 (0.51-3.09)	0.6
	Bone and Lymph Node	0.89 (0.49-1.62)	0.7	0.95 (0.42-2.16)	>0.9
	Any Visceral	1.40 (0.73-2.71)	0.3	0.42 (0.11-1.60)	0.2
	Baseline hemoglobin^†^	0.72 (0.66-0.80)	**<0.001**	0.74 (0.66-0.84)	**<0.001**
	Baseline albumin^†^	0.39 (0.25 – 0.60)	**<0.001**	0.50 (0.27-0.93)	**0.029**
	Baseline ALP ≤ ULN	0.46 (0.29-0.72)	**<0.001**	0.41 (0.21-0.78)	**0.007**
	TP53	1.60 (1.02-2.50)	**0.039**	1.91 (1.05-3.49)	**0.035**
	TMRSS2:ERG	1.51 (0.93-2.46)	0.10	1.47 (0.76-2.82)	0.2
	PTEN	1.59 (0.97-2.60)	0.064	1.50 (0.78-2.87)	0.2
	MYC	2.01 (1.03-3.91)	**0.040**	3.81 (1.81-7.99)	**<0.001**
	RAD21	1.91 (0.98-3.71)	0.056	2.55 (1.13-5.77)	**0.024**
	SPOP	0.93 (0.41-2.15)	0.9	0.31 (0.04-2.23)	0.2
	BRCA2	2.12 (1.02-4.42)	**0.045**	2.10 (0.83-5.36)	0.12
	CTNNB1	1.24 (0.57-2.71)	0.6	0.90 (0.28-2.93)	0.9
	APC	0.33 (0.10-1.06)	0.062	0.41 (0.10-1.69)	0.2
	ATM	0.71 (0.26-1.95)	0.5	0.32 (0.04-2.29)	0.3
**Multivariable analysis**	Race				
	White	-	-		
	Black/African Amer.	1.77 (1.08-2.88)	**0.022**		
	Other	0.33 (0.08-2.88)	0.14		
	ECOG				
	0	-	-	-	-
	1-3	2.47 (1.32-4.65)	**0.005**	1.87 (0.78-4.50)	0.2
	Unknown	1.17 (0.26-5.27)	0.8	0.00 (0.00-Inf)	>0.9
	Lack of pain at ARPI initiation	0.72 (0.41-1.25)	0.2	0.61 (0.28-1.34)	0.2
	Low volume disease	0.59 (0.33-1.06)	0.076	0.53 (0.24-1.18)	0.12
	Liver metastases	1.66 (0.78-3.54)	0.2		
	TP53	1.85 (1.11-3.08)	**0.018**	1.80 (0.92-3.54)	0.087
	MYC	1.25 (0.59-2.64)	0.6	4.41 (1.24-15.7)	**0.022**
	RAD21	-		0.40 (0.10-1.64)	0.2
	BRCA2	1.62 (0.70-3.77)	0.3	-	

^†^analyzed as continuous variables, per point rise in g/dL.

*ALP* alkaline phosphatase, *ULN* = upper limit of normal range (135 IU/L for ALP).

Somatic alterations in *MYC* (PFS HR 2.01, 95% CI 1.03-3.91; OS HR 3.81, 95% CI 1.81-7.99) and *TP53* (PFS HR 1.60, 95% CI 1.02–2.50; OS HR 1.91, 95% CI 1.05–3.49) associated with inferior PFS and OS. *BRCA2* additionally associated with inferior PFS (HR 2.12, 95% CI 1.02–4.42) but not OS, while *RAD21* (HR 2.55, 95% CI 1.13–5.77) additionally associated with poorer OS.

On multivariable analysis of clinical and genomic predictors, Black ancestry (HR 1.77, 95% CI 1.08–2.88), ECOG ≥ 1 (HR 2.47, 95% CI 1.32–4.65) and *TP53* alteration (HR 1.85, 95% CI 1.11–3.08) associated with inferior PFS, while *MYC* amplification remained associated with inferior OS (HR 4.41, 95% CI 1.24–15.7) (Table 3).

### Association with PSA endpoints

Median time to PSA nadir was 6.8 months (95% CI 5.8-7.8), with a median nadir of 0.03 ng/mL (range 0-321). PSA50 and PSA90 response rates were 99% and 95%, respectively. Most patients reached PSA nadir within 6 months (48%; versus 6-9 months [18%], 9-12 months [16%], >12 months [19%]). Overall, 145 (67%) achieved a PSA nadir <0.2 ng/mL (versus 10 [4.6%] 0.2-0.4 ng/mL and 63 [29%] >0.4 ng/mL), most (78%) within 12 months, and this associated with improved PFS (HR 0.14, 95% CI 0.10–0.20) and OS (HR 0.19, 95% CI 0.13–0.29).

Achieving PSA nadir <0.2 ng/mL at 12 months associated with low-volume disease (OR 2.89, 95% CI 1.64-5.18), prior definitive local therapy (radical prostatectomy, prostate radiotherapy) (OR 2.18, 95% CI 1.14–4.30), lack of pain (OR 2.47, 95% CI 1.42-4.36), higher hemoglobin (OR 1.23, 95% CI 1.08–1.42) and albumin levels (OR 2.43, 95% CI 1.32–4.71), and normal alkaline phosphatase (OR 2.81, 95% CI 1.51-5.32). Conversely, ECOG ≥ 1 (OR 0.34, 95% CI 0.19–0.61), de novo metastatic disease (OR 0.49, 95% CI 0.24–0.95), visceral metastases (OR 0.33, 95% CI 0.13–0.83) or both bone and lymph node metastases (OR 0.34, 95% CI 0.15–0.75), had lower odds of achieving PSA nadir <0.2 ng/mL within 12 months. On multivariable analysis, only low-volume disease (OR 2.40, 95% CI 1.06–5.60) associated with PSA nadir <0.2 ng/mL at 12 months, while bone and lymph node metastases associated with decreased odds (OR 0.38, 95%CI 0.15–0.91) (Supplementary Table S1).

*TMPRSS2::ERG* (OR 0.39, 95%CI 0.18-0.83) and ≥1 proliferative gene alteration were associated with failure to achieve PSA nadir <0.2 ng/mL at 12 months (OR 0.49, 95% CI 0.24-0.98), while absence of detectable genetic alterations associated with achieving this nadir (14% vs. 4.1%; OR 3.67, 95% CI 1.09–16.7) (Table 2). *TMPRSS2::ERG* remained independently associated with failure to achieve PSA nadir <0.2 ng/mL within 12 months after adjustment for clinical and disease characteristics (OR 0.37, 95% CI 0.15–0.87).

### Post-progression exploratory analyses

At data cut-off, 148 patients had progressed to APMR disease. Radiographic progression without PSA progression occurred in 30%, associating with *PTEN* loss (OR 2.94, 95% CI 0.85–10.3) and ≥2 TSG alterations (OR 2.61, 95% CI 0.77-10.5) (Supplementary Table S2). At progression, 20% (30/148) met AVPC criteria, including 50% (4/8) of ER compared to 23% (13/56) of NR. AVPC was characterized in ER patients by through biopsy evidence of NEPC (*n* = 1), low PSA with bulky radiographic disease (*n* = 2), and liver metastasis at progression (*n* = 1), while it was evidenced in NR through biopsy (*n* = 5), chromogranin A elevation (*n* = 2), low PSA with bulky disease (*n* = 4), presence of liver metastasis (*n* = 6), and clinical suspicion by treating clinician (*n* = 6) (note patients may have more than one AVPC feature; Supplementary Table S3). Logistic regression analysis among progressed patients with temporal molecular data (*n* = 64) did not identify genetic predictors of AVPC development (Supplementary Table S4). Post-ARPI survival (from time of ARPI progression to death or last follow-up) was longer for ER versus NR (24-month estimated post-ARPI survival 51% ER versus 24% NR) (Supplementary Fig. S4).

## Discussion

In this retrospective analysis of ARPI-treated patients with mAPMS, we characterized the clinical and molecular determinants of exceptional response, survival and post-treatment PSA kinetics in the modern era. Non-responders demonstrated a higher baseline genomic alteration burden compared to exceptional responders, with enrichment in TSG and proliferative genes such as *MYC* amplification. *TP53* mutation/loss and *MYC* gain consistently predicted poorer PFS and OS, while no single alteration predicted achieving PSA nadir <0.2 ng/mL at 12 months. Rather, the absence of poor-risk clinical and genetic features most strongly predicted an undetectable PSA nadir and exceptional long-term responses.

Early, deep PSA responses to ARPI are associated with improved survival in prostate cancer [25]. PSA50 and PSA90 rates were high even among short-term responders, reflecting APMS sensitivity to ARPI/ADT and limiting the discriminatory utility of these broad PSA endpoints. Achieving PSA < 0.2 ng/mL within 6-12 months is prognostic in randomized and real-world cohorts [26–29] and associated with an over five-fold increased likelihood of achieving exceptional response in our cohort. Molecular predictors of this endpoint remain poorly defined. While no individual gene associated with achieving PSA < 0.2 ng/mL at 12 months, proliferative pathway alterations and *TMPRSS2::ERG* fusion were associated with failure to reach this threshold. *TMPRSS2::ERG* fusion is a common, early molecular event [30] promoting androgen-regulated *ERG* overexpression. Despite theoretical increased sensitivity to androgen pathway modulation, its prognostic and predictive significance remains inconsistent [31–33]. Notably, *TMPRSS2::ERG* status was not predictive of PFS nor OS in our dataset.

To further delineate molecular correlates of divergent outcomes, we compared quartile-defined non-responders and exceptional responders. Quartile definitions in this real-world cohort were consistent with randomized trials [4, 10, 12]. Molecular profiling is limited from phase 3 APMS trials; however, post-hoc transcriptomic analyses from TITAN demonstrated that ARPI may mitigate adverse molecular risk for Decipher genomic classifier (GC) high-risk tumors, underscoring the prognostic relevance of baseline profiling [34]. Loss of key TSG (*TP53, PTEN, RB1*) is a recognized driver of ARPI resistance and increases in prevalence as disease progresses [35]. *TP53* alterations were enriched in NR, despite lacking association with early PSA response, potentially reflecting its role in mediating adaptive lineage plasticity and non-PSA based resistance mechanisms [36]. *PTEN* status did not independently associate with response but did associate with radiographic/PSA discordant progression, consistent with the CAPITELLO-281 trial [37]. Given emerging evidence suggesting transcriptomic *PTEN* loss predicts docetaxel sensitivity [38], the prevalence of triplet therapy in our cohort (36%) may have attenuated its independent predictive value for ARPI response. Broader pathway-level definitions incorporating *PI3K* and proliferative genes may therefore be more useful. Co-alteration in ≥1 TSG and proliferative pathway genes was strongly associated with NR, suggesting accounting for co-occurring alterations in predictive models may improve accuracy in mAPMS’ heterogenous landscape [39]. *BRCA2* alterations have also been associated with poorer survival mAPMS [40, 41] and, while more prevalent in NR (15% vs. 0%), associated with inferior PFS but not OS in this analysis. *RAD21* alterations emerged as a predictor of inferior OS, consistent with prior clinical-genomic studies [42] and representing a novel gene of interest.

In contrast, *APC*, a WNT pathway gene, appeared enriched among ER (23% vs. 3%). Despite associations with high-volume disease [43] and visceral metastases at APMR progression [44, 45], WNT alterations are associated with longer time-to-APMR and improved OS, even after adjusting for other high-risk features [20], supporting our findings. *SPOP* alterations, also associated with favorable outcomes [46, 47], were infrequent in our cohort (7%), limiting analysis. An absence of baseline genomic alterations significantly associated with early, deep PSA responses and ER status, while NR demonstrated a significantly higher alteration burden.

Clinical predictors of PSA outcomes and exceptional response were largely consistent, including low volume disease, ECOG 0, higher hemoglobin and albumin, and normal alkaline phosphatase level. Clinical predictors of ER/NR generally demonstrated greater effect sizes compared to genomic factors (e.g. strongest clinical factor OR 12.4 for disease volume versus strongest genomic factor OR 0.19 for *TP53* alteration). Despite association with poor survival in some randomized trials [48], de novo metastatic disease did not predict ARPI response in our cohort, consistent with large sequencing studies of APMS and a meta-analysis of randomized treatment-intensification trials [20, 49]. This may be due to underrepresentation of low-volume and metachronous patients in our cohort, who have excellent outcomes and may not yet have had tumor testing in the absence of APMR progression. Contemporary real-world data demonstrates improving survival for patients with de novo disease, particularly younger populations [50], though relative contributions from intensified treatment paradigms and stage migration due to advanced imaging techniques (e.g. PSMA-PET) remain uncertain.

Study limitations include potential selection bias due to the single-institution design and incomplete data from the real-world electronic medical record. Molecular data was heterogenous by assay type and specimen source and cfDNA assays may have limited sensitivity for detecting certain genomic alterations including structural rearrangements in *TMPRSS2::ERG* and TSG losses, which may influence observed correlations. Molecular data was only available in a subset of patients who likely progressed over time, and due to the small number of observed alterations of some genes (e.g. *BRCA2, SPOP*), may be underpowered to detect survival differences for these alterations. Results should be viewed as preliminary and hypothesis-generating, to guide future studies with pre-specified hypotheses and appropriate adjustment for multiple testing necessary to validate these findings. Despite these limitations, this study comprehensively characterizes the clinical and genomic landscape of mAPMS and factors underpinning PSA kinetics, exceptional responses and survival. Our results reinforce the utility of early molecular testing to complement established clinical factors for prognostication.

## Supplementary information

Supplementary tables

Supplementary Figure 1

Supplementary Figure 2

Supplementary Figure 3

Supplementary Figure 4

## Data Availability

Deidentified clinical and genetic data for external validation of results is available upon request of the corresponding authors under IRB approval.
