# Supplementary material for: Clinical and genomic determinants of extraordinary response and resistance to androgen receptor pathway inhibitors in men with metastatic androgen pathway modulator sensitive (APMS) prostate cancer
Source: Prostate Cancer Prostatic Dis. 2026 Aug 5;29(5):1019–27. doi: 10.1038/s41391-026-01145-0 (PMC13719515; doi:10.1038/s41391-026-01145-0)
Supplement: Supplementary file 1 [file 41391_2026_1145_MOESM1_ESM.docx]

**Supplementary Files**

**Supplementary Methods**

- Definitions of biologic groupings for pathway-level analyses

**Supplementary Tables**

- **Supplementary Table S1.** Clinical predictors of PSA nadir <0.2 ng/mL at 12 months
- **Supplementary Table S2.** Association between discordant progression and genetic alterations
- **Supplementary Table S3.** Evidence of AVPC per ER status
- **Supplementary Table S4.** Association between top 10 genes and aggressive variant prostate cancer

**Supplementary Figure Legends**

- **Supplementary Figure S1.** Consort diagram. Of 154 with temporal molecular data available, 62% had FoundationOne CDx tissue testing (107 primary tissue, 32 metastatic tissue) and 87 (38%) had cfDNA testing (85 Guardant360, 2 FoundationOne LiquidDx). ARPI = androgen receptor pathway inhibitor, mAPMS = metastatic androgen pathway modulation sensitive prostate cancer, mAPMR = metastatic androgen pathway modulation resistant prostate cancer.
- **Supplementary Figure S2.** Oncoprint of genes in >1 patient in the temporal testing cohort. Oncoprint for mAPMS patients with temporal genetic testing showing genes with alterations occurring in >1 patients (n=68 genes). Includes 136 patients with an alteration in any of these genes.
- **Supplementary Figure S3.** Kaplan-Meier plots of progression-free survival and overall survival per exceptional response status (N=224)
- **Supplementary Figure S4.** Kaplan-Meier plot of post-progression survival after ARPI (N=63) based on prior response to an ARPI in the APMS setting

**Supplementary Methods**

Alteration grouping per biologic pathway -

- Tumor suppressor genes (TSG; TP53, PTEN, RB1, CHD1)
- DNA damage and homologous repair genes (BRCA2, BRCA1, ATM, CHEK2, MSH2, MSH6, MSH3, ATR, FANCA/G, ERCC4, PALB2, BARD1, RAD51, CDK12)
- Androgen receptor (mutation and gain - genomic structural rearrangements were not available in the NGS data)
- Proliferative signaling pathways (WNT pathway [APC, CTNNB1, RNF43, AXIN1], RAS/RAF/MAPK pathway [BRAF, FGFR1, FGF, EGFR, HRAS, KRAS, RET, NF1, ERRB3, ERRB4, IRS2, JAK2, JAK3, NRAS, MAP3K], PI3K signaling [PIK3CA, PIK3R1, PI3KC2B, AKT1, INPP4B, STK11, PPP2R2A], MYC, BCL2, BCL2L1, CCN genes [CCND1, CCNE1], CDK8, CDKN, CIC, EP300, ESR1, FAS, GATA4, HGF, LYN, MED12, MLL2)
- Epigenetic regulators (ARID1A, ASXL1, EZH2 and KDM [KDM5A, KDM5C, KDM6A]).

**Supplementary Table S1.** Clinical predictors of PSA nadir <0.2 ng/mL at 12 months (N=224)

|  | **Univariable** | | **Multivariable*** | |
| --- | --- | --- | --- | --- |
| **Characteristic** | **OR (95% CI)** | **p-value** | **OR (95% CI)** | **p-value** |
| Age | 0.99 (0.96, 1.02) | 0.6 |  |  |
| Race  White  Black/African Amer.  Other | -  0.74 (0.39, 1.38)  0.60 (0.15, 2.17) | -  0.3  0.4 |  |  |
| History of smoking | 0.64 (0.37, 1.08) | 0.093 |  |  |
| Gleason Score  6-7  8-10  Unknown | -  1.02 (0.49, 2.13)  0.62 (0.26, 1.47) | -  >0.9  0.3 |  |  |
| ECOG  0  1-3  Unknown | **-**  0.34 (0.19, 0.61)  0.43 (0.17, 1.08) | **-**  **<0.001**  0.074 | -  0.53 (0.25, 1.14)  1.33 (0.25, 7.76) | -  0.10  0.7 |
| Lack of pain at ARPI initiation | 2.47 (1.42, 4.36) | **0.002** | 1.25 (0.59, 2.61) | 0.5 |
| Prior localized treatment | 2.18 (1.14, 4.30) | **0.020** | 1.55 (0.43, 6.01) | 0.5 |
| De novo metastatic | 0.49 (0.24, 0.95) | **0.038** | 1.13 (0.30, 4.40) | 0.9 |
| Low volume disease | 2.89 (1.64, 5.18) | **<0.001** | 2.40 (1.06, 5.60) | **0.039** |
| Metastasis locations  Bone only  Lymph Node only  Bone and Lymph Node  Any Visceral | -  0.73 (0.26, 2.01)  0.34 (0.15, 0.75)  0.33 (0.13, 0.83) | -  **0.021**  **0.009**  0.5 | -  0.38 (0.11, 1.33)  0.38 (0.15, 0.91)  0.59 (0.20, 1.70) | -  0.13  **0.033**  0.3 |
| Median hemoglobin (g/dL) ^†^ | 1.23 (1.08, 1.42) | **0.003** |  |  |
| Median albumin (g/dL) ^†^ | 2.43 (1.32, 4.71) | **0.006** |  |  |
| Alkaline phosphatase ≤ULN | 2.81 (1.51-5.32) | **0.001** |  |  |
| Neutrophil/lymphocyte ratio | 1.02 (0.91, 1.15) | 0.7 |  |  |
| ^†^analyzed as continuous variables, per point rise in g/dL  ULN = upper limit of normal range (135 IU/L for alkaline phosphatase)  Multivariable analysis excludes variables with a high rate of missingness. | | | | |

**Supplementary Table S2.** Association between genetic alterations and discordant progression

| Characteristic | Gene Counts | | Logistic Regression | |
| --- | --- | --- | --- | --- |
|  | **No PSA progression** N = 16*^1^* | **PSA progression** N = 43*^1^* | **OR***^2^* | **95% CI***^2^* |
| TP53 | 8 (50%) | 17 (40%) | 1.53 | 0.48, 4.94 |
| TMPRSS2:ERG | 6 (38%) | 10 (23%) | 1.98 | 0.56, 6.81 |
| PTEN | 7 (44%) | 9 (21%) | 2.94 | 0.85, 10.3 |
| MYC | 1 (6.3%) | 6 (14%) | 0.41 | 0.02, 2.70 |
| RAD21 | 1 (6.3%) | 3 (7.0%) | 0.89 | 0.04, 7.58 |
| SPOP | 1 (6.3%) | 3 (7.0%) | 0.89 | 0.04, 7.58 |
| BRCA2 | 2 (13%) | 3 (7.0%) | 1.90 | 0.23, 12.7 |
| CTNNB1 | 3 (19%) | 3 (7.0%) | 3.08 | 0.52, 18.5 |
| APC | 0 (0%) | 3 (7.0%) |  |  |
| ATM | 0 (0%) | 2 (4.7%) |  |  |
| ≥1 Tumor suppressor genes | 12 (75%) | 23 (53%) | 2.61 | 0.77, 10.5 |
| ≥2 Tumor suppressor genes | 3 (19%) | 3 (7.0%) | 3.08 | 0.52, 18.5 |
| ≥1 WNT pathway | 3 (19%) | 8 (19%) | 1.01 | 0.20, 4.12 |
| ≥1 DNA damage pathway | 2 (13%) | 11 (26%) | 0.42 | 0.06, 1.82 |
| ≥1 RAS/RAF/MAPK pathway | 4 (25%) | 6 (14%) | 2.06 | 0.46, 8.49 |
| ≥1 PI3K signaling | 2 (13%) | 11 (26%) | 0.42 | 0.06, 1.82 |
| DNA damage sensing/repair | 2 (13%) | 11 (26%) | 0.42 | 0.06, 1.82 |
| Proliferative genes | 12 (75%) | 36 (84%) | 0.58 | 0.15, 2.54 |
| Epigenetic genes | 1 (6.3%) | 6 (14%) | 0.41 | 0.02, 2.70 |
| TSG & DNA repair | 2 (13%) | 5 (12%) | 1.09 | 0.14, 5.71 |
| TSG & Proliferative | 9 (56%) | 22 (51%) | 1.23 | 0.39, 4.01 |
| TSG & Epigenetic | 0 (0%) | 4 (9.3%) |  |  |
| DNA repair & Proliferative | 2 (13%) | 11 (26%) | 0.42 | 0.06, 1.82 |
| DNA repair & Epigenetic | 0 (0%) | 4 (9.3%) |  |  |
| Proliferative & Epigenetic | 1 (6.3%) | 6 (14%) | 0.41 | 0.02, 2.70 |
| No genetic alterations | 0 (0%) | 4 (9.3%) |  |  |
| *^1^* n (%); *^2^* OR = Odds Ratio, CI = Confidence Interval | | | | |

**Supplementary Table S3.** AVPC patients per ER/NR status

| **Pt** | **ER/NR** | **Genes altered^*^** | **Evidence of AVPC** | | | | | | **PSA/ radiographic discordance** |
| --- | --- | --- | --- | --- | --- | --- | --- | --- | --- |
|  |  |  | Biopsy (NEPC) | CEA >ULN | ChA >ULN | Low PSA w bulky disease | Liver mets | Clinical suspicion |  |
| 1 | NR | ERBB4, MYC, PIKC3A, FGF12, PRKCI | X |  | X |  |  |  | No |
| 2 | NR | TP53, BRCA2, BRCA1, CHEK2, AKT1  (gBRCA negative) |  |  |  | X |  |  | No |
| 3 | NR | SF3B1 | X |  |  |  | X |  | No |
| 4 | NR | TP53, BRCA2, AKT1 | X |  |  |  | X |  | Yes |
| 5 | NR | TP53, PIK3CA, HRAS |  |  |  | X | X | X | No |
| 6 | NR | TP53, RB1, PTEN, MYC, TMPRSS2:ERG, KRAS, RAD21, CTNNB1 | X |  | X |  |  |  | Yes |
| 7 | NR | MSI-H:  TP53, PTEN, RB1, PIK3CA, ATM, APC, NF1, BRCA2, MSH6, BRAF, CHEK2, CTNNB1, JAK2, FANCA, SMAD4, TSC1  (gBRCA negative)* |  |  |  | X |  |  | Yes |
| 8 | NR | TET2* |  |  |  |  | X |  | No |
| 9 | NR | TP53, AR, CDK6, ARIDA1, PDGFRA* |  |  |  |  | X | X | No |
| 10 | NR | MSI-H:  PTEN, PIK3R1, MSH2, QKI, NTRK1, MAP3K1, HNF1A, CEBPA, FAM123B, FLCN, MLL2, ASXL1* | X |  |  |  |  |  | No |
| 11 | NR | BRCA2, AR, APC*  (gBRCA2 positive) |  |  |  |  |  | X | No |
| 12 | NR | MSI-H  TP53, PTEN, PALB2, ATM, APC, CTNNB1, ARIDA1, FANCA, FGFR1, MET, TSC1* |  |  |  | X |  |  | Yes |
| 13 | NR | CDKN2A, MTAP |  |  |  |  | X |  | No |
| 14 | ER | RB1, PTEN, ATM, MTAP, FAS, BCOR, CDKN2B, CDKN2A, CDKN1B* | X |  |  |  |  |  | Yes |
| 15 | ER | No alterations* |  |  |  |  | X |  | No |
| 16 | ER | RB1, APC, ARIDA1* |  |  |  | X |  |  | Yes |
| 17 | ER | BRAF, KRAS* |  |  |  | X |  |  | Yes |
| AVPC = aggressive-variant prostate cancer, CEA = carcinoembryonic antigen, ChA = chromogranin A, MSI-H = microsatellite instability high/unstable, NEPC = neuroendocrine prostate cancer, ULN = upper limit of normal  *genomic testing performed post progression | | | | | | | | | |

**Supplementary Table S4.** Association between top 10 genes and aggressive variant prostate cancer

| Characteristic | AVPC | | Logistic Regression | |
| --- | --- | --- | --- | --- |
|  | **No N = 58*^1^*** | **Yes N = 26*^1^*** | **OR*^2^*** | **95% CI*^2^*** |
| TP53 | 35 (60%) | 15 (58%) | 0.90 | 0.35, 2.33 |
| AR | 19 (33%) | 6 (23%) | 0.62 | 0.20, 1.72 |
| PTEN | 12 (21%) | 9 (35%) | 2.03 | 0.71, 5.69 |
| PIK3CA | 9 (16%) | 6 (23%) | 1.63 | 0.49, 5.15 |
| TMPRSS2:ERG | 12 (21%) | 1 (3.8%) | 0.15 | 0.01, 0.85 |
| BRCA2 | 8 (14%) | 5 (19%) | 1.49 | 0.41, 5.01 |
| RB1 | 5 (8.6%) | 5 (19%) | 2.52 | 0.64, 9.97 |
| APC | 3 (5.2%) | 5 (19%) | 4.37 | 0.98, 22.8 |
| ATM | 4 (6.9%) | 3 (12%) | 1.76 | 0.33, 8.61 |
| CTNNB1 | 4 (6.9%) | 3 (12%) | 1.76 | 0.33, 8.61 |
| *^1^* n (%);*^2^* OR = Odds Ratio, CI = Confidence Interval. AVPC = aggressive-variant prostate cancer | | | | |
