# Supplementary material for: Clinical and genomic determinants of extraordinary response and resistance to androgen receptor pathway inhibitors in men with metastatic androgen pathway modulator sensitive (APMS) prostate cancer
Source: Prostate Cancer Prostatic Dis. 2026 Aug 5;29(5):1019–27. doi: 10.1038/s41391-026-01145-0 (PMC13719515; doi:10.1038/s41391-026-01145-0)
Supplement: Supplementary file 2 [file 41391_2026_1145_MOESM2_ESM.docx]

**Supplementary Figure S1.** Consort diagram

Of 154 with temporal molecular data available, 62% had FoundationOne CDx tissue testing (107 primary tissue, 32 metastatic tissue) and 87 (38%) had cfDNA testing (85 Guardant360, 2 FoundationOne LiquidDx).

ARPI = androgen receptor pathway inhibitor, mAPMS = metastatic androgen pathway modulation sensitive prostate cancer, mAPMR = metastatic androgen pathway modulation resistant prostate cancer.
